# Supplementary material for: Are carbon emissions trading and green financial instruments synergistic? -Comprehensive quantitative research based on content analysis
Source: PLoS One. 2024 Mar 7;19(3):e0298601. doi: 10.1371/journal.pone.0298601 (PMC10919601; doi:10.1371/journal.pone.0298601)
Supplement: S1 Data — (ZIP) [file pone.0298601.s001.zip › Data/地级市Z_F工作报告.zip!/║■▒▒/╗╞╩»╩╨/└┤╫╘╣1⁄2╓┌║┼í╛┬φ┐╦╩2╛▌═°í┐_2017.docx]

各位代表：

现在，我代表市人民政府，向大会报告政府工作，请予审议，并请市政协各位委员和其他列席人员提出意见。

一、2016年及本届政府工作回顾侯卫东官场笔记

2016年，面对经济下行压力持续加大的严峻态势，在中共黄石市委的坚强领导下，全市上下树立新理念、抢抓新机遇、推动新发展，实现了“十三五”良好开局。初步预计，地区生产总值增长7％，规模以上工业增加值增长8.1％，固定资产投资增长12.5％，地方公共财政预算收入增长5％，外贸出口增长13％，社会消费品零售总额增长12％。

（一）抓产业、扶实体，稳住经济增长

推进产业升级。深入实施工业振兴计划，设立4支、16亿元产业投资基金，支持上达电子、三丰智能装备等一批重点项目，电子信息、装备制造等产业快速发展。发放企业转型和帮扶资金6000多万元，引导企业实施500万元以上技改项目122个。市财政预算和债券资金安排3500万元，支持模具钢产业转型升级。引导成立现代服务业联合会，28个总投资242亿元的服务业重点项目加快实施，服务业增加值增长8.5％。旅游业总收入增长18％。大力发展现代农业，新增规上农产品加工企业17家、农民合作社438家、家庭农场264家，农产品加工业总产值增长10.4％。

扶持实体经济。制定出台金融支持工业企业实施意见等政策文件，设立6亿元中小企业应急循环资金池和1.46亿元生产流动资金，帮助企业完成续贷30亿元。大力开展以“五抓一促”为主题的千名干部进千企活动，盘活武汉重冶等企业，解决了一批问题。实施中小企业成长工程，新增规上企业100家。

扩大有效投资。深入推进产业链招商，新注册重点项目123个，实际到位内资920亿元、增长16％，实际利用外资1.46亿美元、增长14％，争取国家专项建设基金30亿元。强力推进项目建设，汉龙汽车、中兴派能等231个重点项目开工建设，携康电子、筠山风电等227个重点项目竣工投产。

突出创新驱动。新认定高新技术企业26家，全市高新技术产业实现增加值150亿元、增长11％，新冶钢荣获第四届中国工业大奖。深入推进大众创业、万众创新，新增国家级众创空间1家、省级科技企业孵化器2家、科技创业企业150家、市场主体3.4万户。实施科技成果转化55项，登记省级以上重大科技成果81项，发明专利授权量110件。黄石大冶湖高新技术产业园区创建国家级高新区加快推进。率先开通国际直连电路，智慧黄石建设稳步推进。狠抓全国质量强市示范城市创建工作，新增中国驰名商标1件、湖北名牌产品6个，荣获“全国质量魅力城市”。

（二）抓新区、强功能，统筹城乡发展

加强新区建设。大冶湖两座跨湖大桥、黄石至阳新一级路建设进展顺利，月亮山隧道、园博大道、圣明路和庆洪路跨线桥及延长线、大广高速连接线等建成通车，新区骨架全面拉开。园博园、矿博园、生态馆建成开放，奥林匹克体育公园、地质馆、城市综合馆、市民中心、鄂东医疗中心、社会福利中心建设加速推进。大冶湖生态新区核心区地下综合管廊试点启动实施。

推进旧城改造。轨道交通线网规划形成初步成果，城市风貌色彩规划完成修编，城市修补规划编制启动。棚改工作超额完成省定任务，市本级货币化安置率达98.5％。四条迎宾线路、花湖大道、伍家洪大道等重点维修改造工程竣工，柯尔山—白马山公园开园。实施“两湖”连通渠疏浚、青山湖清淤、李家坊隧道涌水治理等十大治水工程，防汛排渍体系不断完善，在“98”特大洪水面前黄石城区没有出现“看海”现象。

完善交通设施。加快新港二期项目建设，散货作业线顺利投产，新港口岸开关运营，新港多式联运示范项目成功获批全国7个培育项目之一。武九客专、棋盘洲长江公路大桥连接线、兴富公路、锦冶公路等项目加快建设，棋盘洲和武穴至富池两座长江公路大桥、沿江大道新港段开工建设，武汉至南昌高速黄石段前期工作启动。新增A级物流企业8家。

推进精准脱贫。全市实现脱贫82个村4.3万人，完成易地搬迁1298户、“三改”4256户，新建旅游扶贫新村16个，精准扶贫样板带和美丽乡村体验带建设成效明显，父子山休闲绿道建成开放，梅溪公路建成通车，大冶市率先实现脱贫。投资8.15亿元，完成各类水利工程2386处。实施高标准农田建设项目14个，建成高标准农田10万亩。全力抓好抗洪救灾工作，改种补种农作物8.87万亩，391户1199间水毁房屋完成重建任务，受灾群众已全部搬入新居。加快推进新型城镇化和美丽乡村建设，阳新县南市村入选中国现代新村，还地桥、陈贵、灵乡入围全国综合实力千强镇，大冶市列入国家新型城镇化试点，并继续入选全国百强县（市）。

（三）抓改革、促开放，加快动能转换

推进供给侧结构性改革。压减粗钢产能73万吨，淘汰落后钢铁产能36万吨，关停模具钢企业165家，关闭煤矿16处、去产能135万吨，三年任务一年完成，全域无落后钢铁产能、无煤炭生产企业。推动棚改货币化安置与房地产市场对接，商品房库存保持在合理区间。制定实施降低企业成本激发市场活力意见，降低企业成本20亿元。成功发行2支企业债券、募集资金44亿元，发行中期票据、短期融资6亿元，获批创建国家社会信用体系建设示范城市。振华化学登陆主板，晨信光电、山力科技登陆新三板，60家企业登陆湖北四板。全国内贸流通体制改革与发展综合试点顺利通过终期评估。成功入选国家物流标准化试点城市。

深化重点领域改革。编制完成市级权力清单和责任清单，推行并联审批、容缺审批和“一枚公章审批”，启动实施“五证合一”、“两证整合”登记制度改革。组建市环境投资公司，市级投融资平台新增资产255.57亿元，新增融资61.94亿元、增长18％。实施市域金融工程，新增贷款100亿元，比年初增长10.6％。激活民间投资，餐厨废弃物处理、开发区新型城镇化等4个项目成功入选国家第三批PPP示范项目。成立鄂东医养集团，县级公立医院改革实现全覆盖。深入推进户籍制度改革，全面放开落户限制。体育产业“管办分离”逐步开展。美尔雅、东贝等企业重组进一步推进。不动产统一登记启动实施。国有农场、农村产权、供销社等改革有序推进。

深入推进对外开放。新增境外投资6600万美元，4家企业实现“走出去”，对外承包工程营业额完成4亿美元，新增外贸出口企业25家，口岸进出口货运量增长18.5％。棋盘洲保税物流中心辐射范围不断扩大，实现进出口货物货值1.64亿元。

（四）抓生态、优环境，推进绿色发展

成功举办首届省园博会、矿博会。经过一年多的精心筹备，首届省园博会、矿博会圆满成功，开园期间，园博园吸引游客60多万人，矿博园交易额超2亿元，引爆了大冶湖生态新区开发建设新热潮。

改善环境质量。认真落实“五水共治”决策部署，河西污水处理厂建成使用，新建污水提升泵站3座、污水管网37公里，疏通排水管网65公里，一门至河西污水处理厂主干管全线贯通，下陆、铁山雨污分流等工程全面启动，铁金港污水直排历史即将结束。狠抓长江沿线码头专项整治，123个非法码头及泊位全部拆除，沿江22家重化工企业得到全面整治。矿山环境恢复治理工程抓紧实施，关闭131家露天采石场，铁山获批全国独立工矿区搬迁改造示范区。扎实开展突出环境问题整治，全年空气优良天数稳步提升。深入开展万家企业节能低碳行动，组织实施烟气脱硝、农业污染源治理等75个减排项目。全面完成大冶湖围网拆除工作。全市万元生产总值能耗比上年下降5。

推进“五城同创”。国家森林城市和国家生态市创建工作不断深入，“绿满黄石”快速推进，造林绿化16万亩，三年任务两年完成，创建省级生态乡镇、村20个。国家卫生城市创建顺利通过国家检查组暗访检查。国家环保模范城市和全国文明城市创建工作稳步推进。

（五）抓民生、兜底线，构建和谐社会

抓好就业和社会保障。积极推进创业带就业工作，城镇新增就业7.4万人，城镇登记失业率2.6％。启动机关事业单位参保登记工作，推进城乡居民医保制度整合，五项社会保险累计参保322万人（次），征缴社会保险费38亿元。城市和农村低保水平居全省前列。

协调发展各项社会事业。市本级民生支出66.73亿元，增长16％。义务教育均衡发展持续推进，5个县（市）区通过国家义务教育均衡发展督导评估验收。湖北师范大学正式挂牌。基本公共卫生服务均等化水平不断提高，农村医疗卫生服务体系逐步完善。全面两孩政策稳妥实施。加快现代公共文化服务体系建设，建成农村文化礼堂300家，建设有线公共免费WＩFＩ热点3000多个。阳新县刘时粘家庭荣获首届全国文明家庭称号。加强工业遗产保护立法，划定“四大工业遗址”文物保护范围和建设控制地带。以筹备省第十五届运动会为契机，加快体育设施建设，国乒基地投入使用，成功举办2016黄石磁湖国际半程马拉松赛、第七届环中国国际公路自行车赛（阳新赛段）等一批赛事，黄石籍运动员刘蕙瑕获得第31届奥运会冠军。

创新社会治理。加强社会治安防控体系建设和网格化管理，抓好“九张网”建设，开展“平安黄石”打击整治行动，群众安全感和治安满意度稳步提升。扎实抓好安全生产工作，安全生产形势总体稳定。食品药品安全工作进一步加强。扎实推进“双拥”工作，荣获省“双拥”模范城称号。

各位代表！2016年各项任务的完成，为本届政府的五年任期划上了圆满句号，黄石经济社会发展取得了重大成就。

——综合实力迈上新台阶。五年来，地区生产总值、规模以上工业增加值、地方公共财政预算收入分别跨越1000亿元、500亿元、100亿元大关，年均增长8.7％、8.8％、15％。

——生态立市拓展新境界。五年来，全市高举生态大旗，开启绿色发展时代，实施“五边三化”生态修复治理工程，建成“八园六带”，万元生产总值能耗和主要污染物减排全面完成省定目标，被评为全国国土资源节约集约模范市，成功列入创建国家生态文明先行示范区、全国新能源示范城市、全国循环经济试点城市。

——产业强市开创新局面。五年来，第三产业占地区生产总值、高新技术产业占工业比重分别提高7.37个百分点和12个百分点，有色金属产业产值突破千亿元大关，黄石正成为全国第三大印刷电路板产业聚集区，华中矿产品交易中心成为全国矿产品贸易中心和定价中心之一。先后被列为全国工业绿色转型发展试点城市、国家新型工业化示范基地、国家信息消费试点城市。

——大城建设拉开新格局。五年来，大冶湖生态新区核心区建设如火如荼，全域黄石发展格局全面展开，全市城镇化率达61.7％，黄石新港正朝着万里长江第一个多式联运港口目标大步迈进。

——改革开放释放新活力。五年来，行政审批制度、投融资体制等改革深入推进，对外开放水平不断提升，市级投融资平台资产规模达900亿元，上市挂牌企业103家，市场主体新增8万多户，进出口总量稳居全省前列，被评为“中国外贸百强城市”。

——民生福祉得到新改善。五年来，城乡常住居民人均可支配收入年均分别增长11％、15％，减少贫困人口8万人，6.6万户棚户区居民圆了安居梦。基本公共服务体系不断完善，覆盖城乡的社会保障体系基本建立。成功创建国家公共文化服务体系示范区，被评为全国创业先进城市、全国社区治理和服务创新实验区、全国“救急难”试点城市，连续五年被评为全省社会治安综合治理优胜市州。

——政府履职能力实现新提升。五年来，着力建设开明、精明、廉明、亲民政府，主动接受人大、政协监督，依法治理能力明显增强，权力运行公开和监督机制基本形成，精神文明和民主法治建设成效显著，社会更加和谐稳定。

同时，审计、统计、物价、外事、侨务、民族、宗教、对台、保密、档案、妇女儿童、残疾人、老龄、援藏、援疆、援利川、国家安全、广播电视、新闻出版、科普、防震减灾、民防、水文、气象、史志等各项工作都取得新成绩。

各位代表！过去五年，黄石的每一天都在成长！五年的成就，是我们全面贯彻落实党和国家路线方针政策的结果，得益于省委、省政府和市委的正确领导，凝聚着全市人民的智慧和汗水，也离不开历届市委、市政府打下的良好基础。在此，我代表市人民政府，向全市人民，向给予政府工作大力支持的人大代表和政协委员，向各民主党派、各人民团体和各界人士，向中央、省直各驻黄单位，向驻黄解放军、武警官兵，向所有关心和支持黄石改革发展的海内外人士，表示衷心的感谢！

回首五年，历程艰辛，经验宝贵：必须始终保持战略定力，深入实施“生态立市产业强市，加快建设现代化特大城市”发展战略，坚定信心、保持耐心、树立雄心，持之以恒推进转型发展；必须坚持应急与谋远相结合，遵循经济规律，积蓄发展动能，主动适应和积极引领经济发展新常态；必须坚持以改革促创新促开放，推进“放管服”改革，发挥市场决定性作用和更好发挥政府作用，着力激发市场主体和全社会的创造力；必须坚持保障和改善民生，推进共建共享，不断提高人民群众的幸福感和获得感；必须加强政府自身建设，强化责任担当，提升依法治理能力。

我们也清醒看到，前进道路上还存在不少困难和问题，主要是：产业转型任重道远，新兴产业还没有形成足够的拉动力，竞争力有待进一步提高；经济下行压力加大，部分企业生产经营比较困难；城市功能还不完善，辐射和带动能力还不够强；脱贫攻坚任务较为繁重，民生改善与群众的期盼还有一定差距；政府系统仍然存在庸懒散作风，行政效能仍需进一步提升。对此，我们将采取有力措施，认真加以解决。

二、今后五年的奋斗目标和工作重点

未来五年，是黄石发展的“足金五年”，是决胜全面小康的历史关口。全市上下务必抢抓机遇、乘势而上、务实重行、埋头苦干，为实现全面建成小康社会第一个百年奋斗目标而不懈努力。

今后五年的总体要求是：高举中国特色社会主义伟大旗帜，以马克思列宁主义、毛泽东思想、邓小平理论、“三个代表”重要思想和科学发展观为指导，深入贯彻习近平总书记系列重要讲话精神和治国理政新理念新思想新战略，按照统筹推进“五位一体”总体布局和协调推进“四个全面”战略布局的要求，深入践行新发展理念，认真落实市十三次党代会精神，深化生态立市产业强市，加快建成鄂东区域性中心城市、长江中游城市群重要战略节点城市和国家生态文明先行示范区，率先在全省全面建成小康社会。

今后五年的奋斗目标是：保持较快经济增长速度和较高投资增长速度，努力实现地区生产总值、规模以上工业增加值、地方公共财政预算收入和高新技术产业增加值年均增长10％以上。城市布局和功能更加优化，大冶湖生态新区核心区形成规模，公共服务水平显著提高，市域一体化格局基本形成，城镇化率超过65％，成功创建全国文明城市、国家卫生城市、国家环保模范城市、国家森林城市和国家生态市。全面深化改革取得新成果，体制机制活力充分释放。法治黄石、平安黄石持续走在全省前列，社会关系更加和谐，社会秩序更加良好，社会局面更加稳定。

今后五年的工作重点是：突出打造先进制造之都、现代港口城市、历史文化名城和宜居运动新城，决胜全面小康，共创美好生活。

——打造先进制造之都。以创建全国老工业基地转型升级示范区为目标，全面对接“中国制造2025”，发展壮大核心产业集群，构建支撑转型发展的现代产业体系；着力完善产业平台，建立产业发展机制，打造“产业公地”；着力打造特钢及延伸加工、铜及延伸加工、电子信息、新能源汽车制造及零部件4个千亿元产业集群，装备制造、生命健康2个500亿元产业集群，新型建材、新能源、纺织服装、精细化工4个200亿元产业集群，建设全国重要的电子信息产业基地、特钢和铜产品精深加工基地、生命健康产业基地、节能环保产业基地。以加快发展新经济为引领，突破性发展现代服务业，着力打造区域性消费中心、现代物流中心、金融中心和大数据中心、科教文化中心、医疗服务中心，建设智慧黄石，服务业占地区生产总值比重达到47％以上。以发展特色农业为重点，推动三次产业融合发展。

——打造现代港口城市。坚持港城联动、以港兴市，以新港“亿吨大港”建设为引擎，抢抓长江经济带和湖北自贸区建设机遇，建成棋盘洲、武穴至富池两座长江大桥，黄咸、鄂咸、武阳三条高速公路，沿江大道及棋盘洲物流园，推进保税物流中心升级为国家综合保税区，着力构建“公铁水管空”衔接无缝化、客运交通便捷化、物流运输智能化的现代化综合交通运输体系，打造区域性综合交通枢纽和长江中游多式联运中心，加快形成以黄石新港、大冶湖生态新区为支撑，沿江城镇为补充的长江中游新兴产业走廊。

——打造历史文化名城。坚持规划引领，注重城市空间立体性、平面协调性、风貌整体性、文脉延续性，优化城市布局形态。加快建设现代公共文化服务先行区。加强文化遗产保护利用，力争矿冶工业遗产列入《世界文化遗产名录》，成功创建国家历史文化名城。推进大冶湖生态新区大开发、大建设、大提升，加快老城区提质升级，推动黄石城区与大冶城区、阳新县城融合对接、一体发展。以举办地学旅游、地矿科普展会为契机，擦亮打响“中国科普圣地、世界地矿名城”名片。深入践行社会主义核心价值观，提高城市文明水平。

——打造宜居运动新城。把修复长江生态环境摆在压倒性位置，强力推进“五水共治”，完善生态文明制度体系，加强生态示范创建，建设天蓝、地绿、水净美丽家园，森林覆盖率超过38％。大力推进创业创新，实现更加充分的就业，不断提高城乡居民收入，着力完善社会保障体系，切实增强人民群众的幸福感和获得感。创新社会治理，维护公共安全，完善治安防控体系，确保社会平安有序、和谐稳定。大力发展“幸福产业”、“银发经济”，全力办好第十五届省运会，推动群众体育、竞技体育、体育产业协调发展，把黄石建设成为省级体育强市和国内知名的运动之城。

三、2017年工作安排

2017年，是喜迎十九大胜利召开之年，也是新一届政府履职的第一年，做好今年的政府工作，意义重大。主要预期目标是：地区生产总值增长8％左右，规模以上工业增加值增长8％，全社会固定资产投资增长12％左右，社会消费品零售总额增长12％左右，地方公共财政预算收入增长8％，外贸出口总额增长9％，居民收入增长与经济增长基本同步。全面完成省下达的节能减排任务。重点抓好以下八项工作：

（一）围绕产业提升价值链、产品迈向中高端，着力振兴黄石制造

提升产业质效。以构建产业价值链为重点，制定出台八大主导产业发展办法，建立产业和企业联动发展机制，加快西普电子、东贝新能源、宝钢黄石新港项目、汉龙汽车零部件等项目建设，培育壮大电子信息、高端装备制造、汽车及零部件等产业。加大对行业领先、全国一流、市场竞争力强产品的政策扶持力度，着力推动产品向消费端、中高端迈进。以传统产业改造提升为重点，实施“百企技改”工程，推进钢铁、有色、建材等产业绿色转型。支持优势企业跨地区、跨行业、跨所有制兼并重组，提高产业集中度。深入实施中小企业成长工程，力争新进规企业100家以上。

加快园区建设。创新园区投融资机制，引导政府投融资平台加大园区基础设施投入，重点建设模具钢、生物医药、智能小车等特色产业园区。加快“退城入园”步伐，引导企业向园区集中，提升园区产业实力。加快园区项目审批、技术研发、环境评价、融资担保等公共服务平台建设，全面提升园区服务水平。坚持错位发展，支持每个县（市）区打造1－2个重点园区，加强重点镇工业小区规划建设，形成一批特色产业基地。

推进创新驱动。大力发展高新技术产业，新增高新技术企业20家以上，高新技术产业增加值增长11以上。力争黄石大冶湖高新技术产业园区创建国家级高新区。加快“两化融合”试点示范企业建设，大力发展大数据产业，打造一批“数字车间”和“智能工厂”。支持重点行业企业形成技术标准联盟，推动产业链、人才链、资金链和创新链融合。深入推进产学研合作，加强知识产权保护和应用，支持新冶钢、劲牌等企业申报国家级工程技术研究中心，完成省级重大科技成果转化项目60个以上，专利申请量、授权量分别突破1300件和1200件。成功创建全国质量强市示范市。

深入开展“双千”活动。全面落实扶持实体经济各项优惠政策，健全“六个一”工作推进机制，强化重大生产力调度，切实解决实体经济发展难题。继续抓好武汉重冶、晟祥铜业等企业盘活企稳，释放最大效益。

（二）围绕新经济这一突破口，着力发展现代服务业

推进以新港多式联运为龙头的现代物流业发展。修编完成《黄石港总体规划》，推进黄石新港二期工程建设，加快实施新港多式联运示范工程项目，率先在全省建成公铁水联运站场，开通一条多式联运示范线路。积极对接湖北自贸区，加快口岸通关能力建设，力争进出口货运量增长10以上。抓好国家物流标准化试点工作，完善保税物流中心配套功能，积极申报综合保税区。推进棋盘洲物流园、罗桥物流园、花湖物流园、下陆公铁联运物流园、铁山秀山峰物流园建设。依托新港加快对接武汉步伐，加强与周边城市的交流协作，主动融入长江经济带一体化发展。

着力发展金融业。深入实施市域金融工程，制定出台全市金融产业发展规划和专项扶持政策，组建金融控股集团，健全融资担保体系，全力服务金融业发展。鼓励新设和引进金融机构，支持金融租赁、融资租赁机构发展。大力支持金融创新，设立黄石市金融创新奖，推动产业金融、普惠金融、绿色金融、科技金融加速发展。加快多层次资本市场建设，鼓励企业采取上市融资、发行债券等方式直接融资。创新财政参股、跟进投资、风险补贴等方式，积极发展股权类投资企业。支持保险、证券等发展壮大。

加快发展全域旅游。整合旅游资源，着力打造以“四大工业遗址”为核心的工业旅游线路，以环磁湖、环大冶湖为重点的都市休闲旅游线路，以仙岛湖、网湖、保安湖、东方山、七峰山、小雷山为重点的生态旅游线路，以父子山、黄荆山、熊家境为重点的养生旅游线路，以鄂东南革命烈士陵园、龙港红军街、大冶兵暴遗址等为重点的红色旅游线路。加快特色镇、特色村建设，发展赏花经济，推进乡村游。编制旅游交通规划。加快实施华新、东钢文化创意产业园等项目，加大工业遗址和历史街区、古民居、古建筑保护力度，加强西塞神舟会、阳新布贴、阳新采茶戏等非物质文化遗产的保护和传承，促进文化旅游融合发展。

大力发展会展经济。按照月月有活动的要求，精心谋划一批会展活动，构建“政府主导、协会承办、实体支撑、市场运作”的会展长效机制。加快铜绿山古铜矿遗址公园建设，完善国家矿山公园、矿博园、地质博物馆、市博物馆“收藏、保护、研究、展示”功能，申报举办首届中国地学旅游大会暨中国（黄石）地矿科普博览会。加快团城山湿地公园等项目建设，积极筹办黄石（下陆）第四届园博会。加强城市形象包装推介，不断扩大黄石的知名度和美誉度。

（三）围绕实现全域脱贫，着力抓好“三农”工作

提升农业产业化水平。围绕打造畜禽、水产、食品饮料3个“百亿元产业链”，大力实施“一村一品、一镇一业”富民工程，加快建设一批10万亩以上特色农产品生产基地。深入实施农产品加工“四个一批”工程，推进大冶现代农产品加工园、棋盘洲现代农产品物流园建设，培育申报2家省级、认定5家市级农业产业化龙头企业，力争农产品加工值突破300亿元。加快阳新富河省级现代农业示范区和国家农业科技园区建设。加快发展农村电商，建设一批“互联网农业”示范园区、示范基地、示范项目。

加强农村基础设施建设。投资9.5亿元，开工各类水利工程2600余处，实施富河综合治理、病险水闸整险、大型泵站更新改造、水毁工程修复、农村河塘清淤等一批重点项目，加固堤防95公里，疏浚河道46公里，除险加固水库18座，新建农村供水工程21处，治理水土流失面积20平方公里。按“四好”标准建设农村公路246公里，完成153个省级贫困村扶贫产业路升级改造。加快中低产田改造和土地复垦利用，完成10万亩高标准农田建设任务。加快农村电网、广播电视有线及通信网络改造升级。提升农业机械化水平。

推进农村生态化整治。突出抓好环父子山开发建设，深入推进“两镇一区”、“两区两带”示范带建设，建成10家市级美丽宜居乡村。加强农业、农村污染防治，加快农村污水、垃圾收集处理项目建设，推进农业“三废”综合利用，着力构建村庄保洁长效机制。

全力打好脱贫攻坚战。坚持“四看”标准，深入实施产业扶贫发展规划，加快实施贫困户致富产业培育工程，引导有劳动能力的贫困人口有序输出，构建新型农业经营主体与贫困户共同发展长效机制。大力开展贫困人口就业、技能培训，力争每个有脱贫能力的贫困户有1个科技明白人、掌握1－2项实用技术。对重度残疾人、事实孤儿、贫困女儿户、孤寡老人等特困人群实行兜底供养。改善农村中小学校、幼儿园办学条件，加大贫困生资助力度，不让一个学生因贫失学。加强标准化乡镇卫生院、村卫生室建设，全面落实贫困村医保补偿政策，免费为贫困人口提供基本公共卫生服务。全面完成易地搬迁和土坯房、厨房、厕所改造任务。

（四）围绕扩大有效投入，着力推进精准招商和项目建设

坚持精准招商。针对主导产业链条上的重点、薄弱点建链、补链、强链，瞄准世界知名、全国领先龙头企业，探索基金招商、股权招商、科技招商、展会招商新模式，着力引进一批行业有影响、市场竞争力强的大项目，力争新注册重点项目150个以上。加大央企、省企招商力度，新签约合作项目20个以上、总投资200亿元以上。精心策划境外招商活动，确保实际利用外资1.5亿美元以上。大力发展总部经济。

加强项目建设。围绕产业转型升级、城市内涵发展、交通体系建设、保障改善民生，高起点、前瞻性谋划储备一批重大项目，做好项目前期工作，推进一批成熟项目开工建设。实施重大项目清单管理、挂牌管理，健全领导包保推进、督办指导、半月通报等机制，构建绿色通道、要素保障、综合服务网络，拓宽项目建设服务载体和平台。定期举行重大项目集中开工、现场观摩拉练活动，力争全年开工和建成重点项目各200个以上。

激活民间投资。认真落实小微企业所得税优惠、阶段性降低“五险一金”等政策措施，实施民营经济倍增计划，精心策划储备和实施一批PPP项目，全面撬动社会资本，力争民间投资增长10％以上。深入推进大众创业、万众创新，新增创新创业平台10家、市场主体2万户以上。

（五）围绕建设区域中心城市，着力推进新型城镇化

加快大冶湖生态新区建设。编制完成核心区实施性规划，完善各类专项规划体系。推进城市综合馆、市民中心、鄂东医疗中心、社会福利中心等重大项目建设，完善新区公共服务功能，推进产城深度融合。制定完善入廊政策，加快实施核心区地下综合管廊项目。完成大冶湖堤防加固工程。开工建设新城大道、经三路、体育南路、滨湖大道西段等“五纵三横”工程，实施奥体景观桥、景观河、景观带等工程，提升新区形象。推进220千伏四连山变电站及园博园500千伏线路迁移工程。支持小城镇建设发展，有序推动农村人口向集镇转移。支持大冶开展国家新型城镇化综合试点。

加快重大基础设施建设。启动连接顺丰机场高速、铁贺省道升级改造前期工作，开工建设武阳高速黄石段、河金省道，加快推进棋盘洲、武穴至富池长江大桥、鄂咸高速、沿江大道阳新段和西塞段、陈贵至还地桥、港湖至保安、木港至洋港等交通项目建设，建成棋盘洲长江大桥连接线、新港铁路支线、黄阳一级公路、沿江大道新港段、铁山至武汉光谷一级公路、武九客运专线，建成一二级公路120公里，大修公路60公里以上。加快推进黄石通用机场规划前期工作，完成沿江大道城区段规划设计。加快城市有轨电车项目建设。建成团城山综合客运站。积极争取国家资本金、PPP项目资金等政策性资金，确保重点设施项目有效投入。

切实抓好旧城提质。加快推进棚户区、城中村和旧城改造，加大货币化安置力度，完成棚户区改造16576户，货币化安置9602套。抓好城市道路、管网、管线建设，加大背街小巷整治力度，推进“海绵城市”建设。实施公交优先战略，优化公交线路布局，提高覆盖面和通达深度。多措并举，着力解决老城区“行车难、停车难、行路难”问题。统筹推进“五城同创”，构建全民参与、城乡联动、整体推进的长效机制，力争成功创建国家卫生城市、全国文明城市。启动健康城市创建工作。提升城市智能化管理水平，抓好垃圾分类处置工作，规范夜市经营，打造整洁有序的市容环境。

（六）围绕供给侧结构性改革，着力增强发展活力

深入推进供给侧结构性改革。坚持去产能与优结构有效结合，强化技术、能耗、环保、安全等硬约束，倒逼模具钢企业达标升级。鼓励优势企业“走出去”，建设海外生产基地，推进产能、产品有效输出。深入推进去库存与棚户区改造、公共租赁住房有效对接，建立房地产市场平稳健康发展长效机制。积极优化政府债务杠杆，降低企业负债率。加大金融信用县（市）创建，防范和打击非法集资、逃废债行为，营造良好金融生态环境。全面落实企业减税、降费、降低要素成本等政策，帮助实体经济度难关、求发展。

加强要素保障。深化投融资改革，用好用活产业基金，加快政府融资平台市场化转型，力争平台融资150亿元以上。将中小企业贷款作为考核金融部门的重要依据，切实解决中小企业“融资难、融资贵”等问题。积极争创全国国土资源节约集约示范市，健全工矿废弃地复垦利用、土地整治、耕地占补平衡、城乡建设用地增减挂钩等机制，加大闲置用地、农村宅基地清理和处置力度，提高征地率、供地率和利用率。加快推进矿业权市场化改革，规范矿产资源勘查开发机制。深入实施“东楚英才计划”，加快技能人才培训基地建设，新增高技能人才4000人以上。

深化其他领域改革。深入推进“放管服”改革，推进经济发达镇行政管理体制改革试点。加强权力清单、责任清单动态管理。完善容缺审批、并联审批、网上联合审批机制。深化“五证合一”“两证整合”登记制度改革，完善企业简易注销登记。全面推行“双随机、一公开”监管模式。推进行政综合执法体制改革试点工作。推进农村土地“三权分置”改革，健全土地流转规范管理机制，构建培育新型经营主体政策扶持体系。加强教育、卫生领域编制备案制管理，稳妥推进生产经营类事业单位转企改制。加快医药卫生体制改革，建立分级诊疗制度。深化工资收入分配改革。抓好国企国资改革。深入创建全国和谐劳动关系综合实验区。推进社会信用体系建设，建立守信激励、失信惩戒机制。

（七）围绕生态文明建设，着力改善人居环境

抓好“五水共治”。落实“五长”责任制，坚持标本兼治，水岸同洁，城乡并进，项目带动，加快实施亚行水污染综合治理项目，推进长江、富河、磁湖、大冶湖、保安湖、网湖等重点流域、重点湖泊生态保护与修复。完善城市污水管网建设十大系统，新建污水管网80公里，完成铁山、新下陆和西塞山新冶钢片区内二、三级管网建设。推进城镇污水处理厂提标升级，全市城镇污水处理厂达到一级A排放标准。

推进节能减排。加强重点领域节能管理，推广应用成熟适用节能技术，抓好重点企业节煤、节电、节水、节材等工作。开展清洁生产转型行动，加快煤炭清洁高效利用。推广应用绿色建筑，新建绿色建筑30万平方米，可再生能源应用55万平方米。严格落实高污染燃料禁燃区规定，实施一批重点减排项目。启动实施20蒸吨燃煤锅炉整治，强化工业废气、机动车尾气、建筑扬尘等专项治理。加大新能源汽车推广应用。

加大植绿护绿。抓好国家森林城市创建工作，深入推进“绿满黄石”行动，抓好荒山荒地绿化、通道绿化、村庄绿化、建成区绿化，完成植树造林16.6万亩，新增和改造城市绿地面积63公顷，创建省级生态乡镇、村30个，省级绿色示范乡村39个。启动国家生态园林城市创建。推进地质环境整治、矿山复绿，完成矿山地质环境治理三期工程，市级绿色矿山达标率30以上，创建绿色矿业发展示范先行区。落实土壤污染综合防治示范区实施方案，推进重金属污染治理和生态修复。

（八）围绕提升人民群众的获得感，着力保障和改善民生

做好精准就业和社会政策托底工作。坚持创业带就业，落实稳岗补贴，抓好建档立卡贫困人员、高校毕业生及就业困难人员就业创业工作，妥善做好去产能职工就业安置，确保城镇登记失业率控制在4.5以内。全面实施机关事业单位养老保险制度，巩固和完善城乡居民基本医疗保险制度，抓好进城落户农民各项保险转移接续工作，积极推动全民参保。按照兜底线、救急难的要求，提高城乡低保和农村五保标准，整合医疗救助、道路交通事故社会救助等基金，建立健全物质资金支持与心理疏导相结合的帮扶体系。加快公办养老机构改革，支持社会力量兴办养老服务机构。加快市残疾人康复中心建设。

大力发展社会事业。优化义务教育资源布局，加大数字化校园达标和示范创建工作，创建现代化学校30所。加快鄂东职教园区建设，调整优化专业设置，推进产教融合、校企合作，培养一批“产业工匠”。完善覆盖学前教育到高等教育的精准助学体系。积极探索建立医保、医疗、医药联动机制，着力整治“大处方”等过度医疗问题，推动医疗服务便民降费提质。支持鄂东医疗集团完善运行机制。积极稳妥落实全面两孩政策，促进人口均衡发展。加强基层综合性文化中心建设，大力开展群众性文化活动。加快发展体育事业，全力做好第十五届省运会筹备及参赛备战工作，积极承办全国、全省大型体育赛事，大力发展山地越野、户外探险等项目，打造户外运动休闲“康谷”。

提升社会治理水平。继续织密“九张网”，加快网格化管理和服务向基层延伸，构建立体化社会治安防控体系。健全多元化纠纷调处机制，建立完善环保、征地拆迁等调解体系。扩大法律援助范围，实现律师法律服务进乡村（社区）全覆盖。保障妇女和未成年人合法权益，做好特殊人群人文关怀。坚持安全生产常抓不懈，营造持续稳定的安全环境。严格食品药品安全监管，严把从农田到餐桌、从实验室到医院的每一道防线。加强应急救援及防灾减灾工作，创建5个省级以上综合减灾示范社区。深入推进“七五”普法。加强国防后备力量建设，支持国防和军队改革，推进军民融合深度发展，落实各项优抚政策，争创全国“双拥”模范城。

各位代表！实现宏伟蓝图，贵在务实重行。我们将肩负历史重任，牢记人民重托，少说多干、真抓实干、埋头苦干，以坚定的信念、优良的作风、务实的态度，敢担当、善作为，全面提升政府治理和服务水平。

坚持依法行政。深入实施《法治政府建设五年工作方案》，健全政府法律顾问、权责清单制度，遵循重大行政决策法定程序，全面提高依法行政能力和水平。加强行政执法监督，规范公正文明执法，推进服务型行政执法。自觉接受人大及其常委会的法律监督和工作监督，自觉接受人民政协的民主监督。扎实推进政务公开，加强政府新闻发布工作，让行政权力在阳光下运行。

强化担当作为。站稳立场，把准方向，牢固树立“四个意识”，全面贯彻落实中央和省、市委各项决策部署，确保令行禁止、政令畅通。直面矛盾敢于担当、优化服务主动担当、依法依规善于担当、认真负责真情担当，勇于担起转型发展主体责任。建立健全容错纠错机制，为担当者担当，为负责者负责，鼓励政府工作人员大胆干事、竞进作为。坚决纠正行政不作为、慢作为、乱作为等现象，着力营造真抓实干、狠抓落实的干事创业浓厚氛围，把确定的工作一件件抓好落实。

坚持高效便民。聚焦重点项目、重点工作，建清单、明责任、定时限，严格执行蓝、黄、红牌管理制度，加强督查问责，提升行政效能。坚持以人民为中心的发展思想，真心实意为人民群众办实事、解难题。坚持便民利民，健全市县乡村四级联动政务服务体系，全面提升为民服务能力和水平。

加强廉政建设。深入推进政府系统全面从严治党，严格落实党风廉政建设主体责任和“一岗双责”，认真执行中央“八项规定”，自觉将纪律和规矩挺在前面。加强行政监察和审计监督。全力支持纪检监察机关工作，坚持反腐无禁区、全覆盖、零容忍，以良好的政风取信于民。

各位代表，新的目标承载新的梦想，新的时代开启新的征程。让我们紧密团结在以习近平同志为核心的党中央周围，在市委的坚强领导下，以功成不必在我的胸襟、功成必须有我的豪气，不忘初心，继续前进，坚定信心，砥砺奋进，为加快迈向现代化特大城市而努力奋斗！
